# Assessment of Renal Measurements and Position in the Syrian Hamster (*Mesocricetus auratus*) Using Survey Radiography and In Situ Macroscopic Anatomy

**DOI:** 10.3390/ani16091298

**Published:** 2026-04-23

**Authors:** Jamal Nourinezhad, Sina Biglary Makvandi, Abdolvahed Moarabi, Mahdi Pourmahdi Borujeni, Sorosh Sabiza, Maciej Janeczek

**Affiliations:** 1 Division of Anatomy and Embryology, Department of Basic Sciences, Faculty of Veterinary Medicine, Shahid Chamran University of Ahvaz, Ahvaz 61357-83151, Iran; 2 Graduated D.V.M. Student of Faculty of Veterinary Medicine, Shahid Chamran University of Ahvaz, Ahvaz 61357-83151, Iran; 3 Division of Radiology, Department of Clinical Sciences, Faculty of Veterinary Medicine, Shahid Chamran University of Ahvaz, Ahvaz 61357-83151, Iran; 4 Department of Food Hygiene, Faculty of Veterinary Medicine, Shahid Chamran University of Ahvaz, Ahvaz 61357-83151, Iran; 5 Division of Surgery, Department of Clinical Sciences, Faculty of Veterinary Medicine, Shahid Chamran University of Ahvaz, Ahvaz 61357-83151, Iran; 6 Division of Animal Anatomy, Department of Biostructure and Animal Physiology, Faculty of Veterinary Medicine, Wroclaw University of Environmental and Life Sciences, 50-375 Wrocław, Poland

**Keywords:** abdominal, exotic companion mammals, diagnostic imaging, measurement, morphology, urinary tract, vertebral body

## Abstract

Although renal disease in Syrian hamsters (SHs) has been reported, imaging studies of normal kidneys in this commonly used pet and laboratory species are lacking, despite the key role of imaging in diagnosis. This study aimed to examine kidneys of Syrian hamsters using radiographic and anatomical methods, focusing on kidney location, visibility, size, and its ratio to the second lumbar vertebra, along with the effects of sex, body size, side, and recumbency. Abdominal radiographs were obtained from 29 clinically healthy adult Syrian hamsters of both sexes to assess kidney visibility, position, and size as well as the length of second lumbar vertebral body on lateral and ventrodorsal (VD) views, followed by an in situ anatomical study for comparative analysis. The kidneys were typically located opposite the first to third lumbar vertebrae. On VD views, the left kidney was generally visible, whereas the right was identified in only 28%. The mean values of radiographic RKL, LKL, and 2LVL were 15.2 mm, 12.44 mm, and 14.27 mm, respectively, and the KL/2LVL ratio ranged from 2.66 to 4.00. No significant sex differences were observed in KL or the KL/2LVL ratio in either anatomical or radiographic measurements (*p* > 0.05). Sex had a significant effect on both radiographic and anatomical 2LVL measurements, with females generally showing higher values than males. Unlike the anatomical measurements, no significant differences between sides were found in radiographic KL and the KL/2LVL ratio. The radiographic RKL, LKL, and 2LVL were significantly larger than those obtained from anatomical measurements. No significant correlation was found between KL, 2LVL, or the KL/2LVL ratio and body length or body weight in either radiographic or anatomical measurements, except for a correlation between body weight and anatomical KL. Right and left kidneys were symmetrically placed, as in rats, but differed from rabbits and guinea pigs. Kidney visibility on VD views was similar to that reported in rabbits. Radiographic RKL, LKL, and 2LVL values differed from those of rodents and rabbits. The radiographic ratio was larger than the values reported in rats, chinchillas, guinea pigs, and rabbits. A single KL-to-2LVL ratio reference range applies to both kidneys and sexes, simplifying clinical assessment.

## Simple Summary

Syrian hamsters were among the most commonly kept companion animals and were widely used as experimental models. However, detailed reference data on normal renal morphology and imaging characteristics were limited. Because kidney size and position could be affected by renal disorders, accurate baseline information was considered essential for diagnostic and prognostic evaluations. Accordingly, the kidneys of clinically healthy Syrian hamsters were examined using complementary radiographic and anatomical approaches, with particular emphasis on renal topography and size. Baseline reference data were provided by the findings, which may support anatomical, radiological, and clinical assessments in veterinary practice and research.

## 1. Introduction

The hamster is one of the most widely used experimental animals, and approximately 90% of hamsters used in research are Syrian hamsters (*Mesocricetus auratus*) [1]. The Syrian hamster is also the most common pet hamster [2]. The most common diseases affecting the kidneys of hamsters include amyloidosis, arteriolar nephrosclerosis, polycystic kidney, and glomerulonephropathy [3].

Imaging is an integral part of the diagnostic evaluation of the renal system [4]. Abdominal radiographs are commonly available and are often the first imaging modality chosen for evaluating the urogenital system in small exotic companion mammals [5]. Radiographs are useful for identifying changes in the number, size, shape, symmetry, location, and opacity of the kidneys [4].

Imaging-based kidney length measurements serve as important clinical indicators of renal function and provide a foundation for diagnosis and treatment [6,7,8,9,10,11,12]. Among these, the radiologic assessment of the kidney length-to-second lumbar vertebral body length ratio has been established as a standardized method for estimating normal renal size, particularly in dogs and cats [4,13]. This technique is now being increasingly applied to small exotic companion mammals as well. Previous studies have investigated the use of renal imaging techniques, such as radiology, in various small mammals—including rabbits [14], chinchillas [15,16], Ferrets [17], guinea pigs [18,19] and rats [20]—and have described renal measurements, location, and visualization. Because radiographic images may be affected by size distortion (magnification), it is recommended to compare the radiographic measurements with the actual size of the organ [4,13]. Such comparisons have been performed in dogs [21] and rabbits [14].

Moreover, a thorough understanding of renal gross anatomy is crucial for accurately interpreting renal radiographs and for comprehending the pathophysiology of various renal diseases.

Currently, no information appears to be available in the literature regarding renal imaging and macroscopic anatomy of Syrian hamster kidneys. Therefore, the objectives of this investigation were defined as follows: (1) to provide the first detailed description of the morpho-topography of the kidneys in Syrian hamsters, (2) to evaluate the visualization of renal shadows in various recumbent positions, (3) to determine renal skeletotopy relative to the spinal column in both radiographs and gross dissections, (4) to measure kidney length and the length of the second lumbar vertebra, to compare these values between radiography and gross dissection, and to determine kidney length relative to vertebral length, and (5) to investigate the influence of sex and body side. This study was conducted in alignment with our previous reports on the gross anatomy and imaging of Syrian hamsters [22,23,24,25,26].

## 2. Materials and Methods

### 2.1. Examined Animals

A total of 29 healthy adult Syrian hamsters were obtained from the Razi Vaccine and Serum Research Institute (Karaj) and the Center of Comparative and Experimental Medicine, Shiraz University of Medical Sciences. The animals were housed in an air-conditioned room and provided with water and standard commercial chow ad libitum, under a 12-h light/dark cycle. The body length (from nose tip to tail base) and body weight of all examined Syrian hamsters were measured and recorded. The age of the hamsters was estimated based on body weight, as reported by Gad [27]. The animals were confirmed to be healthy based on their individual histories and clinical examinations, as well as whole-body radiologic and ultrasonographic evaluations (Z60 Vet, Shenzhen Mindray Animal Medical Technology Co., Shenzhen, China). No clinical complications were observed in any of the examined animals. The hamsters were not fasted prior to radiologic examinations [2,23,26].

### 2.2. Radiographic Acquisition

Immediately before the imaging examination, the hamsters were anesthetized intraperitoneally with a combination of ketamine, acepromazine, and xylazine. The anesthetic mixture consisted of ketamine (50 mg/mL, 5 mL), acepromazine (10 mg/mL, 1 mL), and xylazine (20 mg/mL, 2.5 mL). Each animal received an injection volume of 0.1 mL per 100 g of body weight, corresponding to ketamine 50 mg/kg, acepromazine 10 mg/kg, and xylazine 20 mg/kg as described in our earlier studies [23,26].

Left-to-right lateral and ventrodorsal (VD) abdominal radiographs were obtained for each animal using a portable X-ray (Dongmun-100P, Goyang-si, South Korea) and a Prima CR radiology system (Model: CR-I392, Fujifilm, Tokyo, Japan). Imaging parameters were set at 30 mAs, 40–45 kVp, and a film-focus distance of 100–110 cm. The abdomen was centered on the cassette, with the thoracic limbs extended cranially and the pelvic limbs extended caudally. No grid was used. Radiographs were evaluated on a DICOM workstation by a single observer (A.M.), and kidney shadow visualization as well as their position relative to the vertebral column were recorded.

### 2.3. Gross Anatomical Study 

The hamsters were euthanized immediately after imaging using dissociative agent combinations, in accordance with AVMA guidelines [22,24,25,28]. The skin and abdominal muscles of euthanized hamsters were then removed from the ventral and lateral surfaces to examine the topographic anatomy of the kidneys in situ. Subsequently, the anatomical features were photographed from the right, left, and ventral aspects of the body using a digital camera (Sony ZV-E10, Tokyo, Japan).

### 2.4. Radiologic and Anatomic Measurements

The following linear measurements were taken: (1) Kidney length (the greatest distance between the cranial and caudal poles of the kidney) of right and left sides of body and (2) Second lumbar vertebral body length (measured at the midpoint, parallel to the greatest longitudinal axis of the vertebral body). All direct linear measurements were made by a single observer (S.B.M.) using a Vernier caliper (200 mm; Mitutoyo Corp., Kawasaki, Japan; resolution: 0.05 mm; graduation: 0.05 mm; accuracy: ±0.05 mm). Radiologic measurements were performed by two observers (A.M. and S.B.M.) using a digital calipers 150 mm Mitutoyo, Japan). All radiographic and anatomical parameters were measured three times on three consecutive days, and the mean values were recorded. Radiographic measurements were obtained only from VD radiographs. The ratios of kidney length to the length of the second lumbar vertebra (L2) were calculated using both direct anatomical measurements and radiographic measurements for each hamster. The anatomically derived kidney and L2 lengths were compared with those obtained from radiographs in the present study.

### 2.5. Statistical Analysis

Statistical analyses were performed using IBM SPSS Statistics for Windows (Version 22.0; Armonk, NY, USA: IBM Corp.). Descriptive statistics, including mean, standard deviation (SD), minimum and maximum values, frequency, and relative frequency, were calculated for the obtained data. Left and right kidney lengths on radiologic and anatomic measurements were compared using a paired samples *t*-test. To assess differences among the two repeated measurements of each variable, a repeated-measures ANOVA was used, considering the effects of sex (male vs. female) between subject factors and measurement methods (anatomy vs. radiology) as within-subject variables. Correlations between body weight and body length with right and left kidney lengths, second lumbar vertebral body length, and the kidney length-to-second lumbar vertebral length (2LVL) ratio in radiologic and anatomic measurements were assessed using Pearson correlation coefficients. Correlations between body weight and body length of the examined Syrian hamsters were also calculated. All statistical analyses were considered significant at *p* < 0.05. The results are presented in tables. Statistical analyses were performed using methods described in the following publications [16,20,21].

## 3. Results

**Figure 1:**
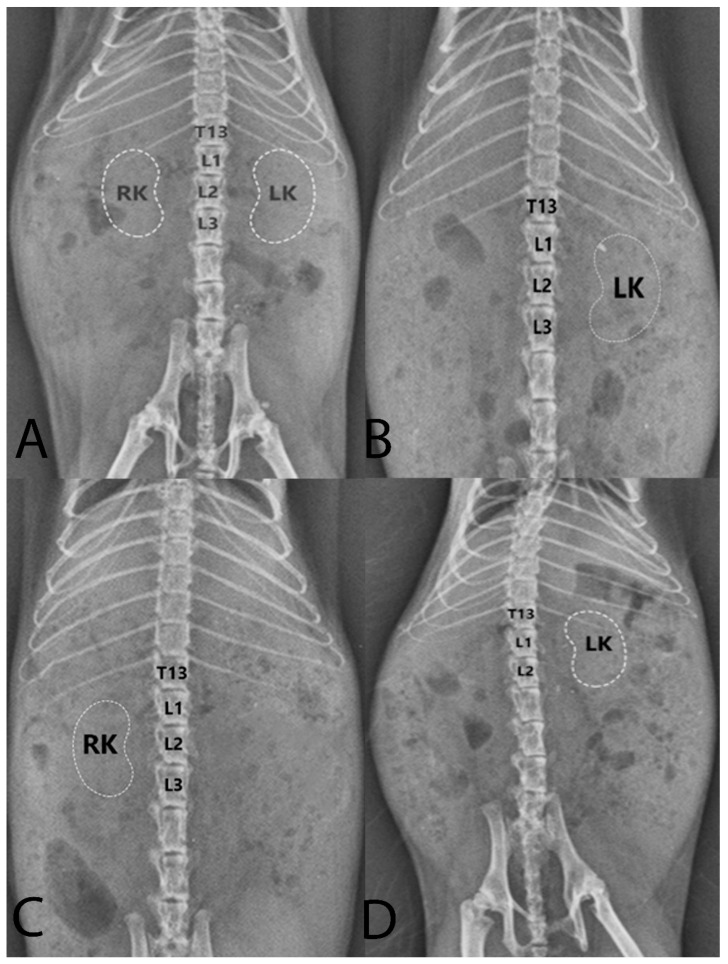
Ventrodorsal radiographs of the Syrian hamster abdomen are shown. In (**A**), the right and left kidneys are positioned symmetrically at the level of the first three lumbar vertebrae. In (**B**), the left kidney is positioned at the level of the first three lumbar vertebrae, but the outline of the right kidney is not visible. In (**C**), the right kidney is located at the level of the first three lumbar vertebrae, but the outline of the left kidney is not visible. In (**D**), the left kidney is positioned opposite the last thoracic vertebra and the first two lumbar vertebrae, but the outline of the right kidney is not visible. T13: thirteenth thoracic vertebra; L1–L3: first to third lumbar vertebrae; LK: left kidney (dashed lines); RK: right kidney (dashed lines).

**Figure 2:**
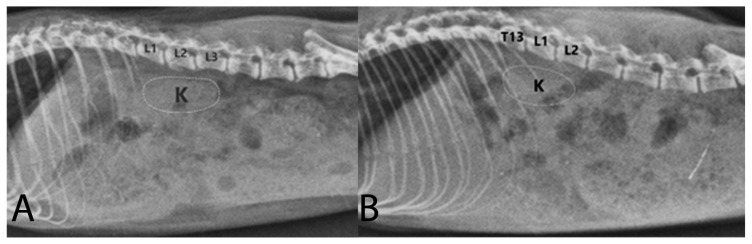
Left lateral radiographs of the Syrian hamster abdomen are shown. In (**A**), the kidney shadows are positioned ventral to the first three lumbar vertebrae. In (**B**), the kidney shadows are located ventral to the last thoracic vertebra and the first two lumbar vertebrae. Due to the symmetrical vertebral positioning of the right and left kidneys, the kidney shadows are nearly perfectly superimposed. L1–L3: first to third lumbar vertebrae; K: kidney (dashed lines).

**Figure 3:**
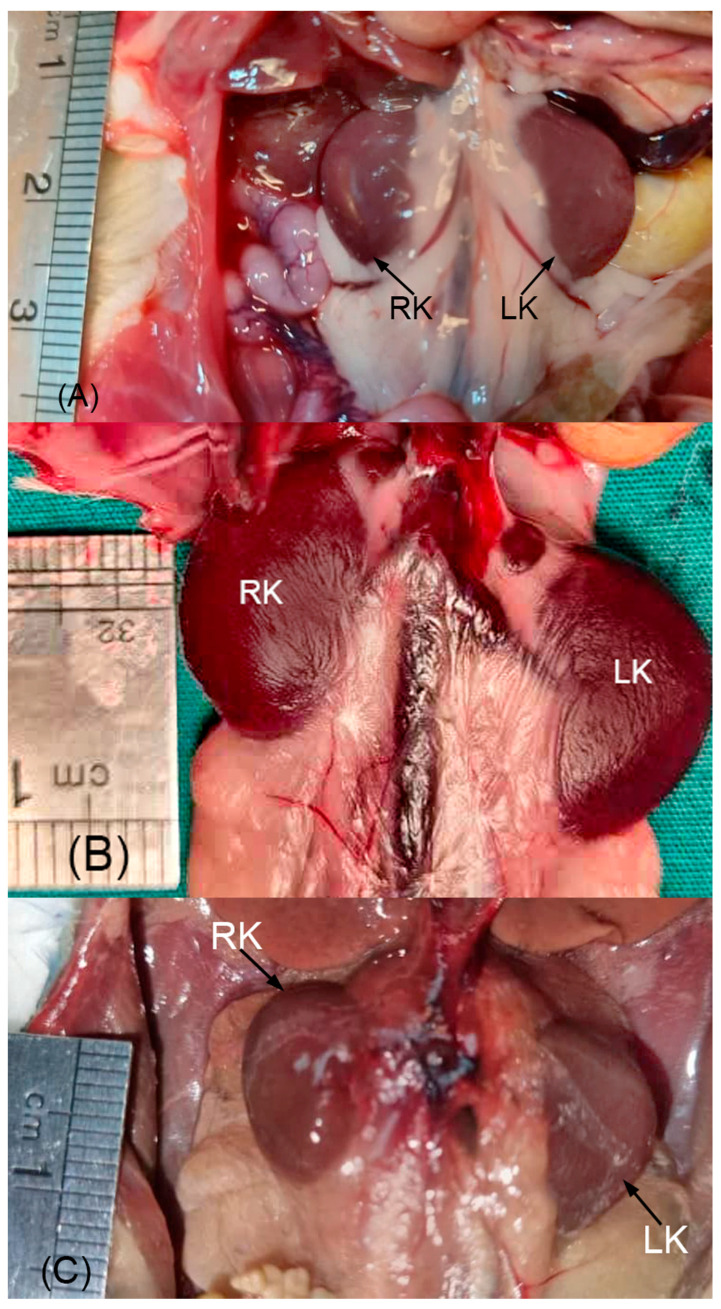
Morphotography of different locations of the Syrian hamster kidneys in situ, ventral aspect, is shown. In (**A**), the hilus of the left kidney is positioned approximately opposite the caudal pole of the right kidney. The ventral surface and lateral border of the kidneys are devoid of perirenal fat. At the renal hilus, perirenal fat is well developed. In (**B**), the right and left kidneys are positioned against the hypaxial muscles and are symmetrically placed. In (**C**), the left kidney is positioned slightly caudal to the right kidney. At the hilus of the left kidney, perirenal fat is well developed, whereas the fat capsule of the right kidney is removed to determine its vertebral position. LK: left kidney; RK: right kidney.

**Figure 4:**
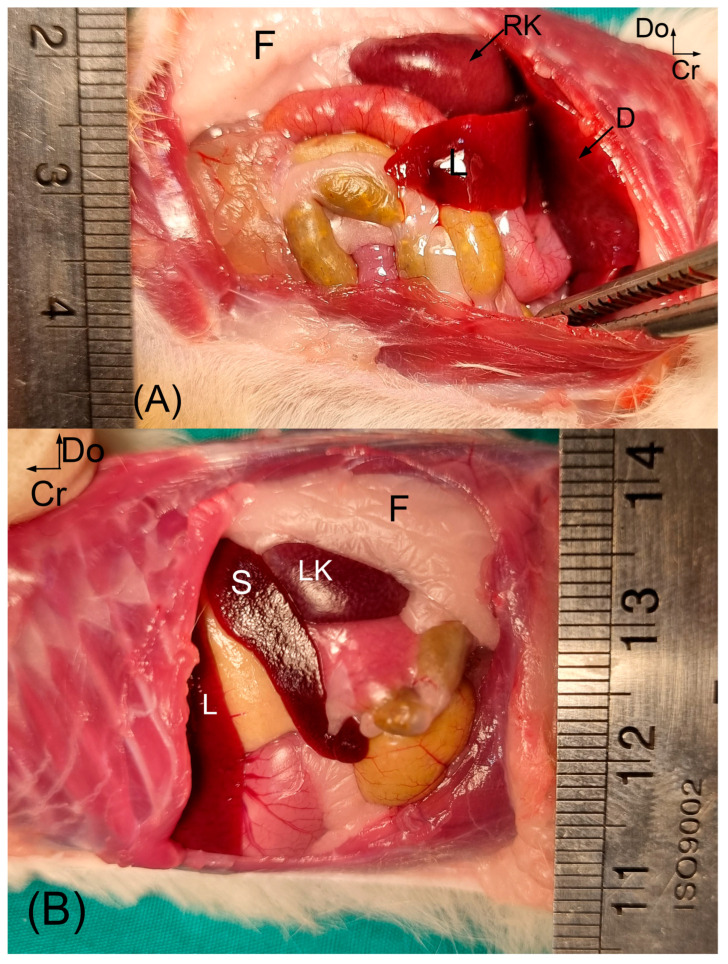
Topography of the Syrian hamster kidney in situ is shown. In the left lateral view (**A**), the cranial border of the left kidney is in contact with the spleen. The dorsolateral surface of the kidney is devoid of perirenal fat. In the right lateral view (**B**), to better identify the syntopy of the kidney, the last three or four ribs are removed, and the diaphragm is displaced cranially and laterally. The cranial border of the right kidney is in contact with the liver and diaphragm. The dorsolateral surface of the right kidney is devoid of perirenal fat. F: perirenal fat; D: diaphragm; L: liver; LK: left kidney; RK: right kidney; S: spleen; Do: dorsal; Cr: cranial.

**Figure 5:**
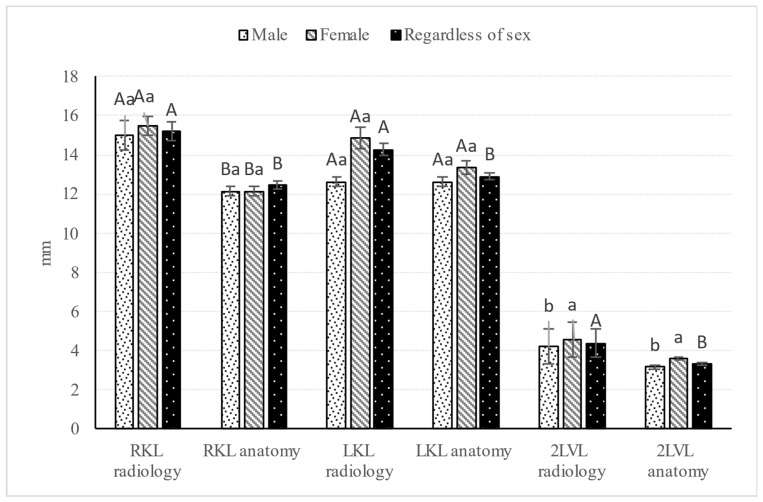
Lengths of the kidney and the second lumbar vertebral body of Syrian hamsters in radiographic and anatomical measurements. Variable notation in the table: uppercase letters (A, B) and lowercase letters (a, b) indicating the effects of side and sex, respectively. Uppercase letters indicating statistically significant differences between radiographic and anatomical measurements, regardless of sex. LKL: left kidney length; LV: Lumbar vertebra; RKL: right kidney length; 2LVL: second lumbar vertebral body length.

**Figure 6:**
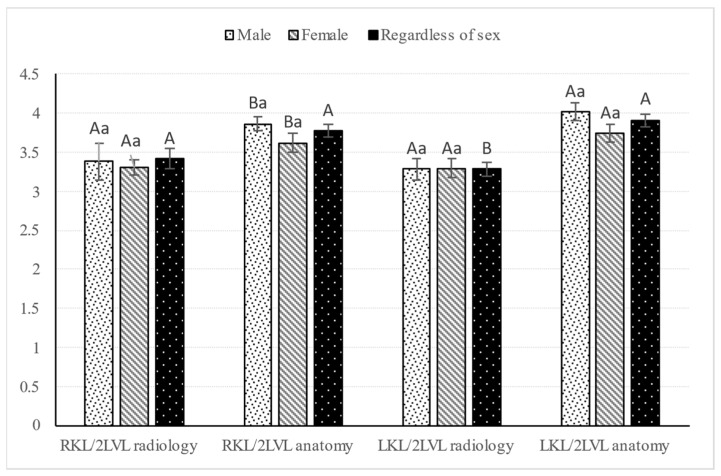
Kidney length-to-second lumbar vertebral body length ratios of Syrian hamsters in radiographic and anatomical measurements. Variable notation in the table: uppercase and lowercase letters indicating the effects of side and sex, respectively, for radiographic and anatomical measurements. RKL: right kidney length; LKL: left kidney length; 2LVL: second lumbar vertebral body length.

Body length and weight of examined Syrian hamsters are presented in Table 1. The visualization of the renal shadow and renal shadow location are shown in Table 2 and Table 3. Ventrodorsal and lateral radiographs of the kidneys are shown in Figure 1 and Figure 2. The in situ topographic anatomy of the kidneys is also illustrated in Figure 3 and Figure 4. Kidney and vertebral body lengths as well as kidney length to 2nd lumbar vertebral body length ratios in radiographic and anatomic measurements are shown in Figure 5 and Figure 6.

On postmortem examination, the kidneys of all hamsters were observed to appear macroscopically normal.

The kidneys were found to be relatively large, bean-shaped, light brown or reddish brown in color, and smooth externally. The right and left kidneys were positioned symmetrically against the hypaxial muscles in the lumbar region, on either side of the median plane (Figure 3). The dorsolateral and ventral surfaces of the right and left kidneys were devoid of perirenal fat, whereas perirenal fat was well developed at the renal hilus. The cranial border of the right kidney was in contact with the liver and diaphragm, while the cranial border of the left kidney was in contact with the spleen (Figure 4).

### 3.1. Length and Weight of Syrian Hamsters (Table 1)

Unlike body length, body weight was shown to have a statistically significant difference between males and females (*p* ≤ 0.05), with greater body weight observed in females than in males. The males were approximately 11 weeks old, and the females were approximately 12–13 weeks old. Body length was found to have a strong and statistically significant positive correlation with body weight (*p* ≤ 0.001).

### 3.2. Number of Lumbar Vertebrae

Six lumbar vertebrae were observed in both male and female Syrian hamsters in each of the 29 individuals studied.

### 3.3. Visualization of Kidney Shadows (Table 2)

On VD views, the left kidney shadow was usually visible in 59% of cases, whereas the right kidney shadow was discernible in only 28% of cases, regardless of sex (Table 2, Figure 1). Both kidneys were outlined on VD views in only 17% of cases, regardless of sex (Table 2, Figure 1A). On lateral radiographs, the right and left kidney shadows were superimposed in 52% of cases, making it impossible to distinguish the two kidneys individually (Table 2, Figure 2). Furthermore, the renal silhouette was not visualized in 48% of cases.

### 3.4. Position of the Right and Left Kidneys Relative to Thoracolumbar Vertebrae in Radiology and Anatomy (Table 3)

On VD views, the right kidneys were most frequently located at the level of the first three lumbar vertebrae in 100% of cases (Table 3, Figure 1). On VD views, the left kidneys were most frequently positioned at the level of the first three lumbar vertebrae in 94% of cases (Table 3, Figure 1). On lateral views, the kidney shadows were most commonly located at the level of the first three lumbar vertebrae in 93% of cases, and the kidneys were superimposed (Table 3, Figure 2). In the anatomical study, the right kidney (69.98%) and the left kidney (82.75%) were most frequently situated at the level of the first three lumbar vertebrae, regardless of sex (Table 3, Figure 3).

### 3.5. Position of the Kidneys Relative to Each Other in Anatomic Study

The position of the right and left kidneys was found to exhibit three distinct patterns. Pattern (1): The kidneys were frequently positioned at nearly the same craniocaudal level (Figure 3B). This pattern was observed in 12 males (41.38%) and 5 females (17.24%), accounting for a total of 17 cases (58.62%), regardless of sex. Pattern (2): The left kidney was positioned only slightly caudal to the right kidney (Figure 3C). This pattern was detected in 6 males (20.68%) and 4 females (13.80%), totaling 10 cases (34.38%). Pattern (3): The left kidney was positioned approximately opposite the caudal pole of the right kidney (Figure 3A). This pattern was found in 1 male (3.45%) and 1 female (3.45%), with an overall frequency of 2 cases (6.90%), regardless of sex.

Among the 29 Syrian hamsters examined, symmetrical and opposite kidney positions were observed in 17 individuals (58.61%), regardless of sex. The right kidney was cranially related to the liver (Figure 4). The left kidney was found to be less firmly fixed than the right and was cranially related to the spleen and diaphragm (Figure 4). Both kidneys were laterally related to the abdominal wall and were palpable through the abdominal wall (Figure 4). In addition, both kidneys were dorsally related to the sublumbar musculature (Figure 3 and Figure 4).

### 3.6. Kidney Length (KL) and 2LVL as Well as KL to 2LVL Ratio in Anatomic and Radiographic Measurements

No significant sex-related differences were found in KL and the KL-to-2LVL ratio in anatomical and radiographic measurements (*p* > 0.05, Figure 5 and Figure 6).

Unlike the radiographic measurements, laterality-related differences in KL and the KL-to-2LVL ratio (*p* ≤ 0.001) were found only in the anatomical measurements, regardless of sex, with the mean LKL and LKL-to-2LVL ratio being greater than the corresponding RKL and RKL-to-2LVL ratio (Figure 5 and Figure 6).

Significant sex-related differences in the 2LVL were found in anatomical (*p* ≤ 0.01) and radiographic (*p* ≤ 0.05) measurements (Figure 5), with mean values observed to be 0.31 mm greater in females than in males.

Significant differences in KL and 2LVL were found between radiographic and anatomical measurements (*p* ≤ 0.001), regardless of sex (Figure 5), with greater values observed in radiological measurements than in anatomical measurements.

The RKL-to-2LVL ratio was not found to differ significantly between radiological and anatomical measurements (*p* > 0.05, Figure 6). However, the LKL-to-2LVL ratio was found to differ significantly between radiological and anatomical measurements (*p* ≤ 0.001, Figure 6), with anatomical measurements observed to be 0.67 times greater than radiological measurements.

In anatomical measurements, significant correlations of the RKL (*p* ≤ 0.001) and LKL (*p* ≤ 0.01) with the 2LVL were found, regardless of sex.

No significant correlations of anatomical or radiological measurements with body length or body weight were found, except that body weight was found to be significantly correlated with the RKL (*p* ≤ 0.001) and LKL (*p* ≤ 0.01) in the anatomical measurements (Figure 6).

In radiographic measurements, the RKL-to-2LVL ratio was found to range from 2.93 to 4.02, regardless of sex; values ranged from 3.08 to 4.47 in females and from 3.25 to 4.48 in males.

In radiographic measurements, the LKL-to-2LVL ratio was found to range from 2.66 to 4.00, regardless of sex; values ranged from 3.40 to 4.75 in females and from 3.28 to 4.98 in males.

## 4. Discussion

Hamsters are adapted to desert environments through both renal and respiratory conservation mechanisms; however, unlike some desert rodents, they cannot survive on metabolic water alone [29].

### 4.1. Imaging Considerations

Diagnostic imaging is essential for assessing renal structural changes, with abdominal radiography being the most accessible first-line modality for urinary system evaluation. In small mammals, whole-body radiographs can often be obtained in a single projection [30]; however, organ visualization is frequently limited due to small organ size and minimal differences in tissue density. Nevertheless, the presence of perirenal fat enhances renal contrast and facilitates kidney identification on radiographs [4,13].

Proper pre-imaging preparation, including gastrointestinal emptying and fasting, improves kidney visibility on radiographs in dogs and cats by reducing intestinal gas [4,13]. Nevertheless, fasting was not implemented for the Syrian hamsters in this study because, like other rodents and rabbits, they are incapable of vomiting [2]. Furthermore, it has been reported that due to the small size and high metabolic rate of laboratory animals, fasting may lead to hypoglycemia and dehydration—even within six hours [31]. Therefore, fasting is not recommended for improving the visibility of kidney shadows in radiographic studies.

### 4.2. Visibility of Kidney Shadows

Two positioning methods are commonly used for kidney radiography in exotic pet animals: ventrodorsal (VD) and lateral recumbency [5,16]. VD recumbency is preferred over dorsoventral (DV) positioning because it allows easier restraint, complete visualization of the abdominal cavity, and improved contrast of retroperitoneal structures [5,13]. In contrast, DV recumbency may cause image distortion due to abdominal compression [4,13]. Accordingly, and consistent with the present findings, VD recumbency is considered the most suitable position for kidney radiography [5].

On the other hand, detecting changes in kidney size is helpful for classifying renal diseases as acute or chronic. In general, assessment of KL is more reliable on VD projections, as there is no overlap of the kidneys, and the magnification of both kidneys is equal [4].

In the present study (29 samples), kidney visualization in the VD view was achieved on the right side in 8 cases (28%), on the left side in 17 cases (59%), and bilaterally in 5 cases (17%), while in the lateral view, kidneys were observed in 15 cases (52%) without distinction between the right and left sides. In chinchillas (28 samples) studied by Karakurm et al. [32], VD visualization occurred on the right side in 3 cases (10%), on the left side in 9 cases (32%), and bilaterally in 3 cases (10%), whereas the lateral view revealed kidneys in 18 cases (64.3%). Dorotea et al. [20] reported VD visualization in laboratory mice (40 samples) on the right side in 19 cases (48%) and on the left side in 24 cases (40%), without data on lateral or bilateral visualization. In dwarf rabbits (19 samples), VD visualization was noted on the right side in 17 cases (89.5%) and on the left side in 18 cases (94.75%), while lateral and bilateral assessments were not performed. Based on the data from various species (hamsters, chinchillas, laboratory mice, and dwarf rabbits), it can be concluded that VD views are preferable to lateral views, as they allow unilateral or bilateral kidney visualization and enable determination of the right and left sides, with higher or more reliable detection rates in most species. In contrast, lateral views have limitations; although kidneys may be visible, the right and left sides are often indistinguishable, and bilateral observation is limited or unclear. Occasionally, the detection rate in lateral views is higher than in VD, but without side assignment, the information is less useful. Additionally, left-sided kidney visualization is more frequent across species, and bilateral visualization has been reported only in some studies, such as in hamsters and chinchillas.

Moreover, bilateral kidney visualization in VD recumbency is generally limited to rabbits, whereas in rodents the visible kidney—if any—is usually the left one, mainly in rats and Syrian hamsters. In chinchillas, poor visualization is attributed to overlapping gastrointestinal contents [16], while in female New Zealand White rabbits it is related to the large cecum [14]. In rats, the absence of pre-imaging fasting results in gastrointestinal distension that hinders kidney detection [20]. In the present study, the right kidney was obscured by the liver shadow and colonic gas, whereas limited visualization of the left kidney may be explained by the large cecum and ascending colon [23].

Based on these observations, in lateral recumbency the right kidney is more readily visualized in chinchillas, whereas the left kidney—despite its more caudal and ventral position—is often obscured by gastrointestinal contents. Consequently, the right kidney is approximately twice as likely to be visible as the left. In the present study, however, symmetrical vertebral positioning and overlapping kidney shadows prevented differentiation between the right and left kidneys.

### 4.3. Kidney Position in Radiographs

In the present study, the kidneys of hamsters were located between LV1 and LV3 on both sides in VD radiographs. Comparative radiographic studies indicate that in White New Zealand rabbits [14], dwarf rabbits [30], and chinchillas [32], the right kidney extends from TV13 to LV3, whereas the left kidney is positioned from LV2 to LV4 in rabbits and from LV3 to LV5 in chinchillas. In guinea pigs, the right kidney lies between TV13 and LV3 and the left between LV1 and LV3 [19]. Overall, the vertebral positions of hamster kidneys are comparable to those of other small mammals, with the kidneys primarily located in the lumbar region and the left kidney generally positioned slightly more caudally than the right.

### 4.4. Kidney Position in Anatomic Study

In this study, the kidneys of Syrian hamsters were located between LV1 and LV3, similar to laboratory rats [33]. Comparable kidney positions have been reported in mole rats (TV13–LV3) [34], chinchillas (LV2–LV4) [35], and gerbils with nearly symmetrical kidneys in the cranial lumbar region [36], whereas guinea pigs and rabbits show a more caudal distribution extending approximately from TV13 to LV5 [37,38,39]. Overall, sexual dimorphism in kidney position is absent in most small mammals, including Syrian hamsters, rats, chinchillas, gerbils, and guinea pigs, but a slight male–female difference has been reported in adult rabbits [39].

### 4.5. KL, 2LVL, and KL to 2LVL Ration as Well as the Influence of the Body Weight in These Variables in Radiographs

In the present study, mean kidney length (KL) in hamsters was 15.2 mm (right) and 14.2 mm (left), with a 2LVL of 4.3 mm, resulting in KL-to-2LVL ratios of 3.47 and 3.77, respectively, which were higher than those reported in other small mammals. In White New Zealand rabbits [14], KL was 30.3 mm (right) and 31.4 mm (left), with ratios of 1.6–2.1 (mean 1.85), while dwarf rabbits [30] showed KL of 21.2 mm (right) and 20.6 mm (left) with ratios around 2.0. In guinea pigs [19], KL measured 22.6 mm (right) and 23.1 mm (left) with a mean ratio of 2.19. Laboratory rats [20] had KL of 36.6 mm (right) and 37.0 mm (left), with ratios ranging from 2.13 to 3.98 (mean 2.13 right, 3.12 left). In chinchillas [32], KL ranged from 14.2 to 22.6 mm with a 2LVL of 8.6 mm, although KL-to-2LVL ratios were not reported. Clinically, deviation of Syrian hamster KL values from these reference ranges may suggest renal pathology.

In Syrian hamsters, body weight was significantly correlated only with left kidney length (LKL), consistent with reports in rats [20], whereas no significant correlation was found with right kidney length (RKL), similar to findings in chinchillas and rats. In contrast, a significant association between body weight and both RKL and LKL has been reported in female New Zealand White rabbits [14], as well as in radiographic studies of dogs [21]. Unlike rats, no significant correlation was observed between body weight and 2LVL in Syrian hamsters or chinchillas.

As in rats [20] and chinchillas [16], the KL-to-2LVL ratio did not show a significant correlation with body weight in Syrian hamsters. Such information has not been reported for female New Zealand White rabbits [14].

Similar to normal captive cheetahs [40], no significant differences were found in KL-to-2LVL ratios between the right and left kidneys. Given the absence of significant associations between this ratio and body weight, body length, or sex, the KL-to-2LVL ratios obtained in this study can be considered reliable and generally applicable. In veterinary radiology, ratio-based measurements reduce the influence of individual factors such as age, sex, breed, and body size, providing more consistent results and improving the detection of renal abnormalities compared with absolute linear measurements.

Although the cranial margin of the right kidney was not clearly visible in all Syrian hamsters, no significant differences were found between right and left kidneys in terms of kidney length or KL-to-2LVL ratios. Furthermore, neither sex, body side, body length, nor body weight had a significant effect on these measurements. Accordingly, a single reference interval for the KL-to-2LVL ratio can be applied to both kidneys in both sexes, enhancing its practical clinical usefulness.

In dogs, the radiographic KL-to-2LVL ratio is higher than the anatomic ratio due to greater kidney magnification; however, considerable individual variation exists, with some dogs showing radiographic ratios similar to anatomic values and others exhibiting marked discrepancies [21]. In Syrian hamsters, no significant difference was found between radiographic and anatomic measurements of the right KL-to-2LVL ratio, supporting equal magnification and the use of a single reference interval. However, for the left kidney, the radiographic ratio was significantly lower than the anatomic value, indicating relatively less kidney magnification compared with the vertebrae.

### 4.6. The Effect of Sex on Measured Variables in Radiographs

In the present study, no statistically significant sex differences were observed in right or left kidney length or in KL-to-2LVL ratios in hamsters, although 2LVL itself differed significantly between sexes. In laboratory rats [20], sex differences were not significant for the right kidney, whereas a weak but significant difference was observed for the left kidney; 2LVL showed no significant sex difference, and the KL-to-2LVL ratio was significant only for the left kidney. Female White New Zealand rabbits [14] showed no significant sex differences in KL, 2LVL, or KL-to-2LVL ratios. Similarly, dwarf rabbits [30] showed no significant sex differences in kidney length or 2LVL, although the kidney-to-vertebra ratio differed significantly between sexes. In guinea pigs [19], sex differences were significant for both right and left kidney length, while data on 2LVL and KL-to-2LVL ratios were not reported. Overall, sex has a limited influence on kidney size and KL-to-2LVL ratios in most small mammals, with guinea pigs being a notable exception. While hamsters, White New Zealand rabbits, and dwarf rabbits generally showed no sex-related differences in kidney dimensions, minor parameter-specific differences were observed in laboratory rats and dwarf rabbits. Hormonal influences related to reproductive status affect kidney length in cats, with intact cats having larger kidneys (2.1–3.2 times L2 length) than neutered cats (1.9–2.6 times L2 length) [4]. Unlike rodents and rabbits, the L2 vertebral length in Syrian hamsters differed significantly between sexes, consistent with findings in domestic ferrets [17].

### 4.7. KL in Anatomic Study

In the present study, mean kidney length (KL) in Syrian hamsters was 12.44 mm (right) and 12.90 mm (left). Comparable values were reported in white rats (12.8 and 12.3 mm) [41]. Larger kidneys were observed in chinchillas (18 mm bilaterally) [35], guinea pigs (18–21 mm right) [37], and laboratory rats (20 mm bilaterally) [24]. Adult rabbits showed the largest kidneys, with right KL of 31.16 mm in adult rabbits [39] and 36.02 mm (right) and 35.84 mm (left) in White New Zealand rabbits [14]. Overall, KL varies markedly among small mammals, with Syrian hamsters and white rats having the smallest kidneys, laboratory rats intermediate sizes, and rabbits the largest, underscoring clear interspecies differences in renal dimensions relevant to comparative studies.

### 4.8. Comparison of KL and KL-to-L2 Ratio in Syrian Hamsters and Small Mammals: Radiographic vs. Anatomical Studies

No significant differences were observed between the right and left kidneys in radiographic measurements, indicating similar kidney size in healthy Syrian hamsters. Additionally, sex had no significant effect on kidney length in either radiographic or anatomical assessments, suggesting comparable renal size in normal male and female Syrian hamsters.

Comparison of anatomic and radiographic kidney length (KL) in Syrian hamsters demonstrated statistically significant differences, with radiographic KL being larger than anatomic KL by a mean of 1.22 mm in the right kidney and 1.10 mm in the left kidney. In rats, a statistically significant difference of 1.27 mm was reported only for the left KL when comparing radiographic and ultrasonographic measurements, and this difference was considered clinically insignificant [20]. Similarly, in a radiographic study on ferrets, a 2 mm difference observed between observers was not regarded as clinically relevant [17]. In New Zealand White female rabbits, radiographic KL exceeded anatomic measurements by 0.83 mm in the left kidney and 0.98 mm in the right kidney, although the statistical significance of these differences was not reported. Taken together, the mean differences observed in Syrian hamsters are comparable to or smaller than those reported in rats, supporting the use of radiographic renal measurements as reliable reference values for Syrian hamsters. The small difference between the mean KL in radiology and anatomy may be due to the use of a tabletop radiology machine and the small size (thin abdominal area) of the animal, which reduces the distance between the kidneys and the film, thereby minimizing magnification. Similar speculations regarding these differences have been made in studies on rats [20] and ferrets [17]. In this regard, previous studies have demonstrated that the distance between the object and the imaging detector significantly affects the degree of image magnification, with an object-to-film distance of 10 cm resulting in approximately 19% magnification [4,13,42]. In addition, the left kidney length in adult dogs has been reported to be 2.98 ± 0.44 times the length of L2 on ventrodorsal views and 2.79 ± 0.46 times the length of L2 on lateral radiographs [43].

On the other hand, the actual kidney size was determined by necropsy, and more precise measurements were obtained using various diagnostic imaging modalities, which is consistent with observations reported in previous studies [21,44].

A limitation of this study is that no biochemical or histopathological evaluations were performed to exclude subclinical renal disease; therefore, the presence of subclinical kidney disease in the Syrian hamsters cannot be ruled out, which may have affected kidney dimensions. Another limitation is the small sample size; a larger number of Syrian hamsters would be needed to establish normal reference intervals. Additionally, the lack of fasting resulted in a large amount of gas in the gastrointestinal tract, which obscured the kidneys and made visualization difficult.

## 5. Conclusions

This study presents the first integrated anatomical and radiographic characterization of kidney position in Syrian hamsters and proposes preliminary reference values for kidney length (KL) and the KL/2LVL ratio. These data provide a foundational framework for renal imaging assessment in this species. Nevertheless, given the limited sample size, the proposed values should be interpreted as preliminary reference benchmarks rather than definitive diagnostic criteria, and further studies with larger cohorts are required to validate and refine these reference ranges.:

## Figures and Tables

**Table 1:** Body length (mm) and weight (g) of male and female Syrian hamsters.

Variable	Sex	Number	Mean	SD	Maximum	Minimum
Body length	Male	18 ^a^	158.66	0.91	166	151
	female	11 ^a^	162	2.4	173	147
	Regardless of sex	29	159.93	1.09	173	147
Body weight	Male	18 ^b^	97.94	1.90	117	85
	Female	11 ^a^	110.36	7.36	145	71
	Regardless of sex	29	102.65	3.16	145	71

Different lowercase letters (^a^, ^b^) indicate significant differences among variables.

**Table 2:** Visualization of male and female Syrian hamster kidneys on two radiographic views.

Views	Side	Male	Female	Regardless of Sex
		N (%)	N (%)	N (%)
VD	Right	5 (17.0)	3 (11.0)	8 (28.0)
	Left	9 (31.0)	8 (28.0)	17 (59.0)
	Both	3 (10.0)	2 (7.0)	5(17.0)
Lateral	Left lateral	10 (35.0)	5 (17.0)	15 (52.0)

VD: Ventrodorsal.

**Table 3:** Vertebral position of the male and female Syrian hamster kidneys in anatomy and two radiographic views.

Vertebral Position	Study	Side	Male	Female	Regardless of the Sex
			N (%)	N (%)	N (%)
LV 1 to LV 3	VD radiograph	Right	5 (63.0)	3 (73.0)	8 (100.0)
		Left	9 (53.0)	7 (41.0)	16 (94.0)
	Anatomy	Right	12 (41.36)	8 (27.62)	20 (69.98)
		Left	16 (55.17)	8 (27.58)	24 (82.75)
TV 13 to LV 2	VD radiograph	Right	0 (0.0)	0 (0.0)	0 (0.0)
		Left	0 (0.0)	1 (6.0)	1 (6.0)
	Anatomy	Right	2 (6.90)	1 (3.44)	3 (10.34)
		Left	2 (6.90)	2 (6.90)	4 (13.80)
LV 1 to LV 4 LV 1 to LV 3 TV 13 to LV 2	VD radiograph	0 (0.0)	0 (0.0)	0 (0.0)	0 (0.0)
	Anatomy Lateral radiograph	Right	4 (13.78)	2 (6.90)	6 (20.68)
		Left	0 (0.0) 10 (67.0) 0 (0.0)	1 (3.45) 4 (26.0) 1 (7.0)	1 (3.45) 14 (93.0) 1 (7.0)

LV: Lumbar vertebrae; TV: Thoracic vertebra.

## Data Availability

Data sharing is not applicable to this article as all data associated is available in the text.

## Abbreviations

KL	Kidney length
LKL	Left kidney length
LV	Lumbar vertebra
RKL	Right kidney length
SHs	Syrian hamsters
TV	Thoracic vertebra
VD	Ventrodorsal
2LVL	Second lumbar vertebral body length
